# Broadening the Phenotypic Spectrum of *MAFB*-Related Disease: Renal, Auricular, Ocular, and Nervous System Involvement

**DOI:** 10.3390/genes17030342

**Published:** 2026-03-19

**Authors:** Aviva Eliyahu, Danit Atias-Varon, Ortal Barel, Yulia Khavkin, Elon Pras, Haike Reznik-Wolf, Odelia Chorin, Tomer Poleg, Ari Biller, Pazit Beckerman, Nabil Abu-Amer, Tamara Wygnanski-Jaffe, Lior Greenbaum, Asaf Vivante, Irit Krause

**Affiliations:** 1 The Danek Gertner Institute of Human Genetics, Sheba Medical Center, Tel-Hashomer, Ramat Gan 52621, Israel; aviva.eliyahu@sheba.gov.il (A.E.);; 2 Gray Faculty of Medical and Health Sciences, Tel-Aviv University, Tel-Aviv 69978, Israel; 3 Genetic Kidney Disease Research Laboratory, Edmond and Lily Safra Children’s Hospital, Sheba Medical Center, Tel-Hashomer, Ramat Gan 52621, Israel; 4 The Genomic Unit, Sheba Cancer Research Center, Sheba Medical Center, Tel-Hashomer, Ramat Gan 52621, Israel; 5 The Morris Kahn Laboratory of Human Genetics, Faculty of Health Sciences, Ben Gurion University of the Negev, Beer-Sheva 84105, Israel; 6 Institute of Nephrology and Hypertension, Sheba Medical Center, Tel-Hashomer, Ramat Gan 52621, Israel; 7 Goldschlager Eye Institute, Sheba Medical Center, Tel-Hashomer, Ramat Gan 52621, Israel; 8 Department of Pediatrics B, Edmond and Lily Safra Children’s Hospital, Sheba Medical Center, Tel-Hashomer, Ramat Gan 52621, Israel; 9 The Talpiot Medical Leadership Program, Sheba Medical Center, Tel-Hashomer, Ramat Gan 52621, Israel; 10 Pediatric Department C, Schneider Children’s Medical Center of Israel, Petah Tikva 4920235, Israel

**Keywords:** *MAFB*, focal segmental glomerulosclerosis, Duane retraction syndrome, chronic kidney disease

## Abstract

Background: Focal segmental glomerulosclerosis (FSGS) is a leading cause of renal disease presenting with steroid-resistant nephrotic syndrome (SNRS) and variable stages of chronic kidney disease (CKD). Monogenic etiologies for FSGS are increasingly recognized, particularly in pediatric and familial cases. Missense variants in the MAF BZIP Transcription Factor B (*MAFB*) gene cause a dominantly inherited condition with variable phenotype, ranging from isolated ocular or renal manifestations to syndromic FSGS. Methods: Detailed clinical and genetic investigations were conducted in an extended family presenting with a spectrum of renal and extra-renal manifestations. Results: Using Exome Sequencing (ES), a heterozygous variant, c.797T>C; p.(Leu266Pro) in the *MAFB* gene was identified in multiple affected family members. Variant segregation confirmed its presence in additional family members. The proband exhibited CKD accompanied by congenital auricular anomalies, hearing loss, and neurodevelopmental delay. An affected sibling presented with nephrotic-range proteinuria, Duane retraction syndrome (DRS) and neurodevelopmental involvement, while another family member had an isolated renal phenotype. Several of these features have not been previously associated with *MAFB.* Tools for structural modeling and stability predictions supported the functional impact of this variant. Conclusions: Our findings expand the phenotypic spectrum of *MAFB*-associated disease and further emphasize its variability.

## 1. Introduction

Focal segmental glomerulosclerosis (FSGS) is a major cause of steroid-resistant nephrotic syndrome (SRNS) and a leading contributor to chronic kidney disease (CKD) in both children and adults [1]. The etiology of FSGS is heterogeneous, involving a complex interplay between genetic predisposition and environmental factors [2]. Over the past decade, however, monogenic causes of FSGS have been increasingly recognized, particularly in pediatric and familial cases [3,4]

To date, pathogenic variants in more than 50 genes have been implicated in monogenic forms of FSGS [5,6]. These genes encode proteins critical for slit diaphragm integrity, cytoskeletal organization, foot process architecture, and the maintenance of podocyte homeostasis [5].

Although FSGS most commonly presents as an isolated renal disorder, a subset of affected individuals exhibits syndromic manifestations, which involve extra-renal organ systems and reflect the broader biological roles of genes essential for podocyte function.

Pathogenic variants in MAF BZIP Transcription Factor B (*MAFB*) (OMIM 608968) are primarily associated with two Mendelian phenotypes: multicentric carpotarsal osteolysis syndrome (MCTO; OMIM #166300), characterized by progressive skeletal osteolysis with possible renal involvement, and Duane retraction syndrome 3 (DRS3; OMIM #617041), a congenital ocular cranial dysinnervation disorder that may co-occur with proteinuric kidney disease.

Recently, heterozygous missense variants in the *MAFB* gene were reported in four individuals from two unrelated families presenting with FSGS in association with DRS, characterized by restricted horizontal eye movements resulting from congenital abnormal cranial nerve development. Additionally, one affected individual exhibited hearing impairment [7]. These observations suggested a role for *MAFB* in the coordinated development and function of renal and neuro-ocular systems.

MAFB is a member of the large Maf family of basic leucine zipper (bZIP) transcription factors [8]. It is expressed in multiple cell types, including macrophages, glomerular podocytes, and osteoclasts, where it plays essential roles in cellular differentiation and tissue-specific gene regulation [9,10,11]. Structurally, MAFB consists of an N-terminal transactivation domain and a conserved C-terminal bZIP domain. The bZIP region comprises a basic segment responsible for sequence-specific DNA binding and an adjacent leucine zipper that mediates dimerization, enabling MAFB to function as a homo- or heterodimer [11].

The biological role of *MAFB* has been further elucidated through studies in animal models, which provide insight into its developmental functions. Beyond renal development, vertebrate models have demonstrated conserved roles for *MAFB* orthologs in hindbrain and cranial motor neuron development. Recently, Jurgens et al. (2025) showed that disruption of the zebrafish *MAFB* ortholog, *mafba*, results in severe malformation or absence of the abducens cranial nerve (CN6) motor nucleus, supporting its evolutionarily conserved role in cranial motor neuron specification [12].

Experimental studies in *Mafb*-deficient mouse models have demonstrated that MAFB is indispensable for podocyte differentiation, foot process formation, and maintenance of the glomerular filtration barrier, underscoring its critical role in renal development and function [13]. A knock-in mouse model harboring the *Mafb* p.Leu239Pro variant within the DNA-binding domain recapitulated multiple developmental abnormalities, including defects of the inner ear, kidney, parathyroid, pancreas, and macrophage lineages, with phenotypic features closely resembling *Mafb*-null mice, further supporting the essential functional role of the bZIP DNA-binding domain [14].

Notably, all missense *MAFB* variants previously associated with FSGS and DRS cluster within or adjacent to the DNA-binding region of the bZIP domain, supporting the hypothesis that impaired transcriptional regulation underlies the combined renal and neuro-ocular phenotype [7]. In contrast, pathogenic variants clustering within the N-terminal transactivation domain cause MCTO, a skeletal-dominant disorder characterized by progressive osteolysis of the carpal and tarsal bones and variable renal involvement, but lacking the characteristic ocular motility defects observed in DRS, thereby highlighting domain-specific genotype–phenotype correlations [15]

In the present study, we describe a family with multiple affected individuals harboring a rare heterozygous variant c.797T>C; p.(Leu266Pro) in *MAFB*, presenting with variably expressed renal involvement, accompanied by incomplete penetrant extra-renal features. This report further expands the phenotypic spectrum associated with *MAFB*-related disease and emphasizes the marked clinical heterogeneity and diagnostic challenges associated with *MAFB* variation.

## 2. Materials and Methods

### 2.1. Clinical Evaluation and Patient Recruitment

Family members were referred for a genetic evaluation and workup at the Nephrogenetic Clinic at the Edmond and Lily Safra Children’s Hospital, Sheba Medical Center, Israel. The referral indication was a family history of CKD, which affected multiple family members.

Demographic and clinical data were collected, including results of previous blood tests and imaging. Blood samples were obtained for DNA extraction and genetic analysis. The study has been approved by the Sheba Medical Center Institutional Review Board. Informed, written consent was obtained by the study participants or by their legal guardians, for genetic analysis and for publication of patients’ photographs.

### 2.2. Exome Sequencing and Bioinformatics Analysis

ES libraries were prepared using the Twist Human Core Exome Plus Kit (Twist Bioscience, San Francisco, CA, USA) and sequenced on an Illumina NovaSeq 6000 platform (San Diego, CA, USA), generating paired-end reads of 150 bp. Sequence alignment to the human reference genome (hg38) was performed using the Illumina DRAGEN Bio-IT Platform (version 3.8), which applies a Smith–Waterman-based alignment algorithm [16]. Variant calling was conducted using the GATK variant caller (version 3.7) [17], with additional variant calling using FreeBayes (version 1.2.0) [18]. Copy number variation analysis (CNV) was performed using DRAGEN pipeline. Annotation of identified variants was carried out using KGG-Seq (version 1.2) [19], followed by further in-house annotation and filtering tools. Variant interpretation and classification were performed according to the American College of Medical Genetics and Genomics (ACMG) guidelines [20].

Sanger sequencing and next-generation sequencing were performed for additional family members as part of a commercial sequencing service.

### 2.3. Variant Curation and Domain Classification

Previously reported *MAFB* missense variants were identified through a systematic search of the Human Gene Mutation Database (HGMD^®^ Professional, version 2025.4) and cross-referenced with the primary literature [21]. Variants were categorized according to their location within functional protein domains (N-terminal transactivation domain versus C-terminal basic leucine zipper [bZIP] domain) and their associated clinical phenotype.

Variants located within the N-terminal transactivation domain and previously associated with multicentric carpotarsal osteolysis (MCTO) were included for comparative domain analysis. Variants within the C-terminal bZIP domain associated with renal and/or ocular phenotypes were further evaluated by structural modeling to assess potential effects on DNA binding or dimerization. Variants lacking a consistent genotype–phenotype correlation were excluded from structural modeling.

### 2.4. Protein/Mutation Modeling Methods

Three-dimensional coordinates of the wild-type protein were obtained from the Protein Data Bank using the crystal structure PDB ID: 2WTY. AlphaMissense software (https://alphamissense.hegelab.org, 10 December 2025) [22] was employed to assess the pathogenicity of the variant. Protein visualization was performed using PyMOL Molecular Graphics System (version 3.1.6) [23]. Predicted change in Gibbs free energy (ΔΔG) was calculated with FoldX5 [24]. Multiple sequence alignments of the *MAFB* sequences were performed using Clustal Omega (https://www.ebi.ac.uk/jdispatcher/msa/clustalo, 10 December 2025) [25]. To visualize and analyze the coiled-coil domains within MAFB, helical wheel diagrams were generated using DrawCoil 1.0 (https://grigoryanlab.org/drawcoil/, 10 December 2025).

## 3. Results

### 3.1. Clinical Characteristics

#### 3.1.1. Patient III-1

**Figure 1:**
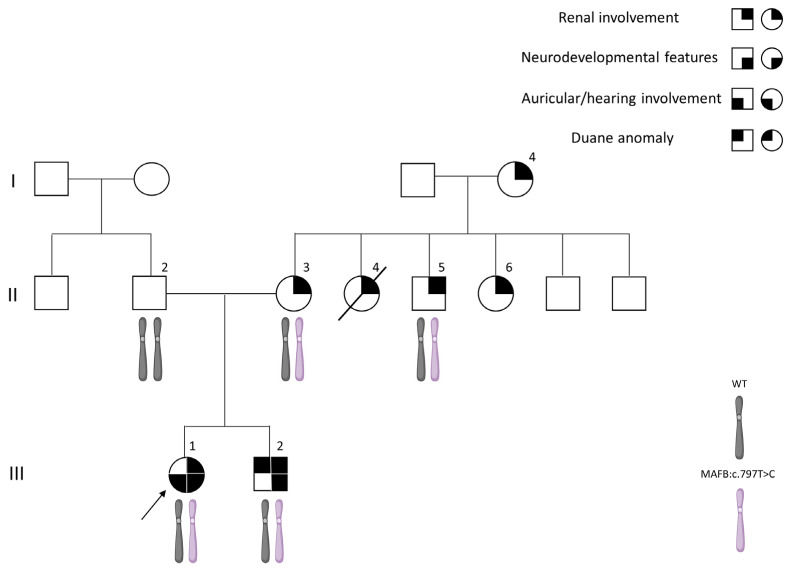
Pedigree of the family harboring the heterozygous *MAFB* c.797T>C; p. Leu266Pro) variant. Clinical manifestations are indicated by phenotype-specific symbols (see key within figure). The arrow denotes the index case. Genotype status is shown for individuals who underwent molecular testing. Roman numerals indicate generations and numbers identify individuals within each generation. The arrow indicates the proband. A diagonal line across a symbol denotes a deceased individual.

**Figure 2:**
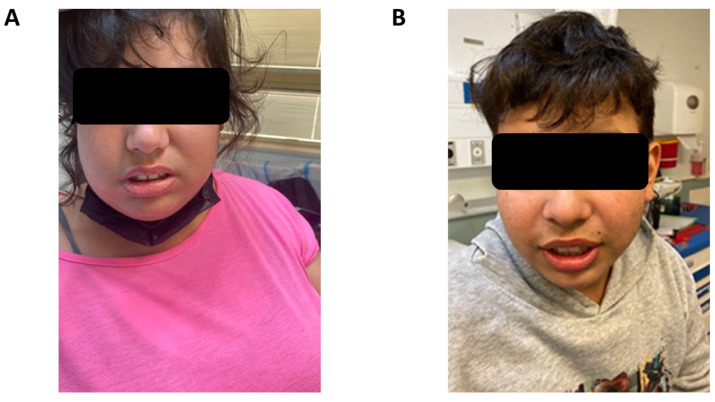
Two affected siblings (Patients III-1 and III-2) harboring the *MAFB* c.797T>C variant. Both siblings (**A**,**B**), display similar dysmorphic features, including a round face, malar flattening, small eyes, a prominent nose, a short philtrum, and small ears.

The proband is a 20-year-old female of Kurdish-Persian Jewish descent (Figure 1). Her medical history is notable for global developmental delay with severe cognitive impairment, features consistent with autism spectrum disorder (ASD), and profound pre-lingual bilateral hearing impairment diagnosed at seven months of age. Brain magnetic resonance imaging (MRI) at one year revealed cochlear aplasia with a bilateral common cavity configuration, without additional intracranial abnormalities. She subsequently underwent bilateral cochlear implantation. Ophthalmological examination was not feasible due to limited patient cooperation related to behavioral and emotional difficulties. Additional medical history included obesity (BMI 47) and pre-diabetes. Physical examination revealed facial dysmorphic features, including a round face, malar flattening, small palpebral fissures, a prominent nose, short philtrum, and small ears (Figure 2A). Laboratory evaluation at age 20 demonstrated abnormal renal function and nephrotic-range proteinuria. Renal ultrasonography showed bilaterally small kidneys with cortical thinning. A kidney biopsy was not performed. Within months, the patient progressed to end-stage kidney disease (ESKD), requiring initiation of maintenance hemodialysis, followed by renal transplantation.

#### 3.1.2. Patient III-2

The proband’s brother is a 17-year-old male who was evaluated for proteinuria due to a family history of CKD. He had nephrotic-range proteinuria (6.7 g/24 h) with a preserved estimated glomerular filtration rate (eGFR). Renal ultrasonography and audiological assessment were unremarkable, and a kidney biopsy was not performed.

The patient’s developmental history included early developmental delay and attention-deficit/hyperactivity disorder (ADHD), and they had attended a special education program. Physical examination revealed obesity (BMI 32) and facial dysmorphic features similar to those seen in his sister. Ophthalmological evaluation demonstrated a severe bilateral abduction deficit, consistent with DRS type 1 (Figure 2B).

#### 3.1.3. Patient II-3

Patient II-3, the 47-year-old mother of Patients III-1 and III-2, was born to consanguineous parents. She was diagnosed with CKD and proteinuria (1200 mg/24 h) at 16 years of age. A kidney biopsy at that time was consistent with FSGS. She was treated with angiotensin-converting enzyme (ACE) inhibitors for several years, during which her kidney function remained stable, with an eGFR of 110 mL/min/1.73 m^2^. This patient denied a history of hearing impairment, ocular abnormalities, or neurodevelopmental disorders.

Two siblings of patient II-3 (Patients II-4 and II-5) were also diagnosed with FSGS and subsequently progressed to ESKD, as described below.

#### 3.1.4. Patient II-4

Patient II-4 developed ESKD at 23 years of age and initiated renal replacement therapy. She underwent successful cadaveric kidney transplantation at age 29. Her neurodevelopmental history was reported as normal. She passed away at the age of 42 years due to cardiac failure. Genetic testing was not performed.

#### 3.1.5. Patient II-5

Patient II-5 progressed to ESKD at 41 years of age and was treated with hemodialysis. At age 43, he received a successful kidney transplant from a healthy sibling (Patient II-6).

#### 3.1.6. Patient II-6

Patient II-6 exhibited low-grade proteinuria but maintained normal kidney function. She served as the living kidney donor for Patient II-5.

#### 3.1.7. Patient I-4

The mother of Patient II-3 (grandmother of Patients III-1 and III-2) was reportedly diagnosed with multiple myeloma later in life, followed by progressive clinical deterioration that included renal failure requiring dialysis at the age of 75 years. Medical records regarding her renal disease or other systemic findings were not available.

### 3.2. Genetic Analysis

ES performed on the index patient, her brother and mother (Patients III-1, III-2 and II-3) identified a heterozygous missense variant in *MAFB* (NM_005461.5): c.797T>C; p.(Leu266Pro). This variant was reported in our previous work summarizing the diagnostic yield of genetic testing within the Nephrogenetic Clinic cohort [26]. However, the initial report only provided a description of the variant with minimal data about its clinical presentation, notably lacking detailed phenotypic characterization and comprehensive familial genetic and clinical investigation.

This c.797T>C; p.(Leu266Pro) variant is absent from population databases (gnomAD frequency -0), and in silico prediction tools uniformly predict a deleterious effect of this substitution (CADD 32, REVEL 0.984). Segregation analysis using Sanger sequencing and/or next generation sequence (NGS) analysis demonstrated maternal inheritance of the variant, which was shared by both affected siblings and by an additional maternal aunt (Patient II-6).

This variant was classified as likely pathogenic variant according to the American College of Medical Genetics and Genomics (ACMG) guidelines [20] (criteria PP1, PM2, PP3, PP4). Although the z score of *MAFB* is moderate, missense variation is a known mechanism of disease with multiple reports of disease-causing missense variants within this gene.

A summary of the clinical, genetic, and segregation findings is provided in Table 1.

Unfortunately, additional samples from maternal family members were not available for further testing.

Given the neurodevelopmental features observed in Patients III-1 and III-2, chromosomal microarray analysis (CMA) was performed and with benign results in both. Molecular testing for CGG trinucleotide repeat expansion at the *FMR1* locus was negative.

No additional pathogenic variants were identified on ES of Patients II-3, III-1, and III-2 that could account for their clinical presentation.

An additional variant in the *ACTN* gene was reported by ES in patients II-3 and III-2 and in segregation analysis in patient II-6 as well. This variant (NM_004924.6): c.448A>G, p.(Ile150Val) is absent from population databases, and in silico prediction tools predict a deleterious effect of this substitution (CADD 25, REVEL 0.83). However, this variant did not segregate within the family and was absent from the most severely affected family member (Patient III-1). According to the ACMG guidelines [20] this variant was classified as a variant of unknown significance (criteria PM2, PP3, PP2, BS4).

**Figure 3:**
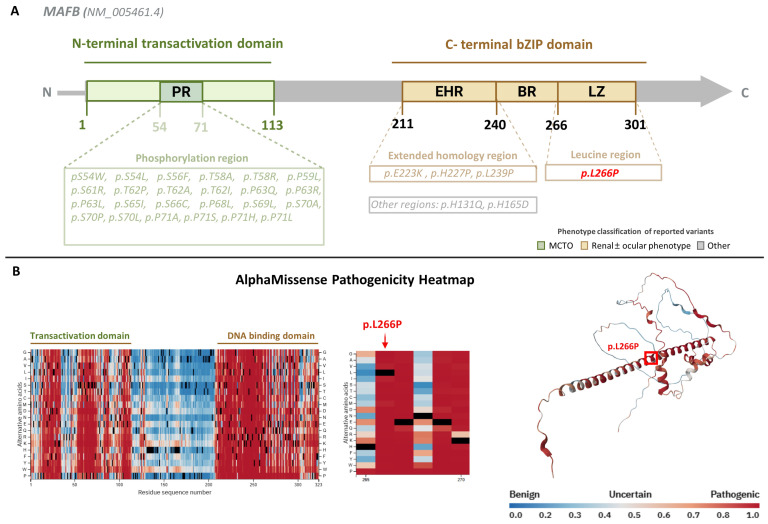
Domain distribution, phenotype classification, and predicted pathogenicity of *MAFB* missense variants. (**A**) Schematic representation of the human *MAFB* protein (NM_005461.4) illustrating its major functional domains and the distribution of previously reported missense variants. The N-terminal transactivation domain (TAD) is shown in green and includes a phosphorylation-rich region (PR). The C-terminal basic leucine zipper (bZIP) domain is shown in brown and comprises the extended homology region (EHR), basic region (BR), and leucine zipper (LZ). Previously reported disease-associated missense variants curated from the Human Gene Mutation Database (HGMD Professional, version 2025.4) and primary literature are indicated according to their domain location. Variant labels are color-coded based on associated clinical phenotype (renal ± ocular phenotype, multicentric carpotarsal osteolysis [MCTO], or other/variable presentations). The variant identified in this study, p.Leu266Pro, located within the leucine zipper region, is highlighted in red. (**B**) AlphaMissense pathogenicity heatmap of the MAFB protein, displaying predicted pathogenicity scores for all possible missense substitutions across the protein sequence. Two regions with consistently high pathogenicity scores (red) are evident and correspond to the transactivation domain and the DNA-binding domain. A zoomed-in view of the region surrounding residue 266 shows that substitutions at this position are predicted to be highly deleterious. Substitutions from leucine to other amino acids have a mean pathogenicity score of approximately 0.99, and the p. Leu266Pro variant reaches the maximal predicted pathogenicity score of 1.0. The right panel shows the AlphaFold-predicted structure of MAFB, with Leu266 highlighted, demonstrating its position within the leucine zipper helix of the DNA-binding domain. Color legend: AlphaMissense pathogenicity scores are displayed on a continuous scale from blue (benign) through light blue/white (uncertain) to red (pathogenic), with values ranging from 0.0 to 1.0, as indicated in the color bar. Created with BioRender.com.

Previously reported *MAFB* missense variants are clustered according to protein domain and associated phenotype. Variants associated with MCTO are located within the N-terminal transactivation domain, whereas variants associated with renal and/or ocular phenotypes are localized predominantly to the C-terminal bZIP domain. Consistently with the domain-specific clustering, the p.(Leu266Pro) variant identified in the present family is localized within this C-terminal region (Figure 3A).

### 3.3. Structural Modeling and Stability Prediction

We next examined the structural consequences of p.(Leu266Pro) in the context of the C-terminal bZIP domain. AlphaMissense pathogenicity mapping demonstrated two major constraint hotspots corresponding to the N-terminal transactivation domain and the C-terminal DNA-binding domain, with residue 266 located within a region of highly predicted pathogenicity (Figure 3B).

**Figure 4:**
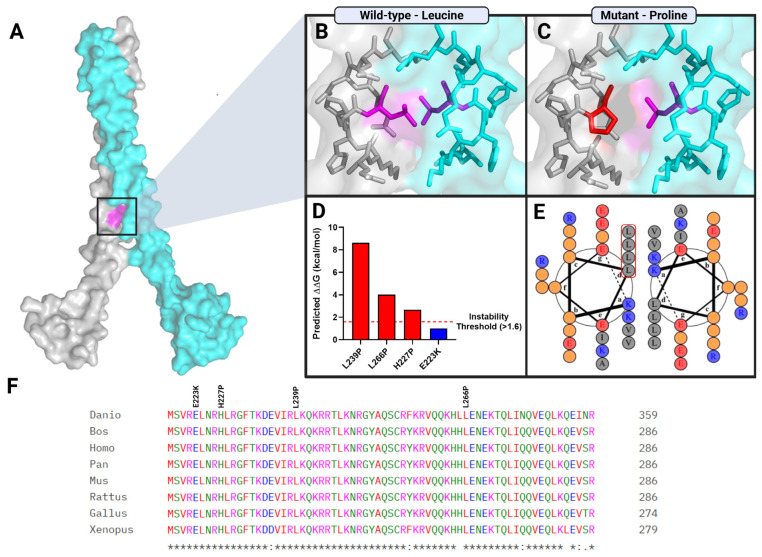
Structural and evolutionary analysis of the MAFB p.(Leu266Pro) variant. (**A**) AlphaFold-predicted structure of the MAFB dimer. Chain A is shown in gray and Chain B in cyan. Residue Leu266 is highlighted in magenta, and the boxed region indicates the leucine zipper interface within the C-terminal bZIP domain. (**B**,**C**) Zoomed-in structural comparison of the leucine zipper region. (**B**) Wild-type configuration showing leucine–leucine interactions at the dimer interface. (**C**) The p. (Leu266Pro) substitution (red) demonstrates altered side-chain geometry within the α-helical coiled-coil. (**D**) Predicted change in Gibbs free energy (ΔΔG, kcal/mol) for selected C-terminal variants. Red bars represent variants predicted to exceed the structural instability threshold (ΔΔG > 1.6 kcal/mol). The blue bar indicates the variant identified in this study, which falls below this threshold. The dashed red line denotes the instability threshold. The p.(Leu266Pro) variant shows marked predicted destabilization relative to other variants. (**E**) Helical wheel representation of the coiled-coil heptad repeat illustrating that residue 266 occupies the conserved ‘d’ position within the hydrophobic core of the leucine zipper. (**F**) Multiple sequence alignment of representative vertebrate *MAFB* orthologs demonstrating strong evolutionary conservation of residues affected by reported C-terminal variants, including Leu266. Created with BioRender.com.

Structural modeling localized Leu266 to the leucine zipper (LZ) segment of the bZIP domain at the MAFB dimer interface (Figure 3A and Figure 4A). In the wild-type configuration, Leu266 participates in the hydrophobic core of the coiled-coil structure, contributing to interhelical packing stability (Figure 4B). Substitution with proline introduces a helix-disrupting residue lacking the aliphatic side chain required for hydrophobic packing, resulting in distortion of local α-helical geometry at the dimer interface (Figure 4C).

Stability prediction analysis further supported this structural perturbation, yielding a ΔΔG of approximately +4.0 kcal/mol for L266P, well above the predefined instability threshold of >1.6 kcal/mol. This indicates a marked predicted destabilization relative to wild type (Figure 4D). Helical wheel projection confirmed the positioning of Leu266 within the hydrophobic heptad repeat core of the leucine zipper (Figure 4E), and cross-species alignment demonstrated strong evolutionary conservation of this residue across vertebrates (Figure 4F), supporting its structural and functional importance. Notably, other residues affected by disease-associated C-terminal variants also exhibit high evolutionary conservation, underscoring the structural and functional constraint of this domain.

## 4. Discussion

In this study, we provide a comprehensive clinical description of a *MAFB* variant, c.797T>C; p.(Leu266Pro), associated with familial FSGS and CKD accompanied by DRS, auricular abnormalities, and neurodevelopmental involvement. Overall, affected family members demonstrated variable clinical expressivity and evidence of reduced penetrance, consistent with previously reported *MAFB*-related phenotypes [7,27]. In patient II-6, a presentation of low range proteinuria is the only reported manifestation. Moreover, since additional family members did not undergo clinical or genetic evaluation, the accuracy of the phenotypic spectrum description is limited.

To date, approximately 30 disease-causing variants in *MAFB* have been reported, several within cohorts presenting with combined renal and ocular abnormalities. MAFB is one of multiple transcription factors essential for podocyte differentiation, particularly in the terminal stages of maturation, leading to the formation of a functional glomerulus [28]. Podocyte-specific *Mafb* knockout studies have shown reduced expression of key podocyte genes—including *Nphs1*, *Magi2*, and *Tcf21*—all required for maintaining the integrity of the slit diaphragm barrier. Variants in these genes are known to cause monogenic FSGS. These findings suggest that in addition to its direct role in podocyte differentiation, *MAFB* may regulate a broader transcriptional network critical for glomerular protein barrier maintenance [13].

The ocular findings observed in our cohort are consistent with the established role of *MAFB* in cranial motor neuron development and the pathogenesis of ocular congenital cranial dysinnervation disorders, as supported by prior murine models and recent functional work by Jürgens et al. [12,29].

MAFB protein also plays a role in ear development. *Mafb* knockout mice demonstrate loss of dorsal otic structures, cochlea and sensory organs. Perturbation of the MAFB–hindbrain pathway influences expression of critical otic genes, including *Gbx2*, *Dlx5*, *Wnt2b*, and *Otx2* [30].

Park et al. (2016) proposed a threshold model in which partial reduction in *MAFB* function leads to isolated DRS, whereas lower levels (e.g., homozygous knockout or dominant-negative effect) result in combined DRS and inner ear abnormalities, indicating tissue-specific sensitivity [31].

A prior report by Pascollini et al. [27] described a family with *MAFB*-associated disease without renal involvement, including one individual with neurodevelopmental delay [27]. In the present study, comprehensive genetic evaluation of multiple family members with neurologic involvement did not identify an alternative cause for the neurodevelopmental phenotype. We therefore postulate that this variant may contribute to the observed neurodevelopmental abnormalities and suggest that such features could represent part of the expanding spectrum of *MAFB*-related disease. However, further studies will be required to establish a definitive association between this gene and the neurodevelopmental phenotype. Although limited information exists regarding the role of *MAFB* in the human brain, experimental evidence demonstrates its involvement in neuronal maturation and microglial function. Loss of *MAFB* expression in macrophage-lineage cells leads to impaired microglial programming and disruption of brain homeostasis [32], and additional work shows that *MAFB* promotes maturation of cortical interneurons [33].

These experimental findings underscore the pleiotropic and tissue-specific functions of *MAFB* in neural development and homeostasis. In this context, accumulating evidence supports a protein domain–dependent model of MAFB pathogenicity. *MAFB* variants affecting distinct functional domains cause clinically discrete yet partially overlapping phenotypes. Missense variants within the N-terminal transactivation domain cause MCTO, a skeletal-dominant disorder that may include craniofacial anomalies and renal involvement but lacks the characteristic ocular motility defects seen in DRS [15]. In contrast, variants associated with ocular cranial dysinnervation and proteinuric kidney disease, including the variant described here, localize to the C-terminal DNA-binding (bZIP) domain (Figure 3A,B).

The p.(Leu266Pro) variant resides within the leucine zipper region of this domain, a critical segment required for dimerization and transcriptional regulation. In silico pathogenicity analysis using AlphaMissense provides structural support for domain-specific functional vulnerability within MAFB. Structural modeling showed that substitution of leucine with proline is predicted to disrupt hydrophobic packing within the heptad repeat core and introduce conformational constraints incompatible with α-helical stability, resulting in marked destabilization of the bZIP dimer interface, as reflected by a ΔΔG value exceeding the predefined instability threshold (Figure 4B–D).

Notably, prior structural analysis of the nearby *MAFB* p.Leu239Pro variant—also located within the DNA-binding region—similarly predicted destabilization of the C-terminal domain [7]. Furthermore, a knock-in mouse model harboring the corresponding *Mafb* p.Leu239Pro substitution exhibited a phenotype closely resembling *Mafb*-null animals, indicating profound functional impairment [14]. Together, these observations support the concept that structural destabilization of the C-terminal bZIP domain represents a shared pathogenic mechanism among non-MCTO *MAFB* variants.

Although our in silico structural modeling predicts only modest destabilization for *MAFB* p.(Glu223Lys), Jürgens et al. demonstrated reduced DNA-binding capacity of this variant using protein-binding microarrays, suggesting that functional impairment may arise from altered transcription factor–DNA interactions.

Conservation analysis further demonstrates strong evolutionary constraint across the C-terminal domain, particularly at residues affected by disease-associated variants (Figure 4F). Collectively, these findings reinforce the domain-dependent functional vulnerability of MAFB and provide a structural framework for the phenotypic segregation observed across its disease spectrum.

Interestingly, although MCTO typically presents with predominantly skeletal manifestations and renal disease is often delayed, partial phenotypic crossover is observed, indicating that domain-specific effects on MAFB function may intersect with tissue-specific thresholds of dosage and genetic background. At present, the reasons for why some individuals develop combined bone–kidney disease while others show isolated organ involvement are not yet clear. Mechanisms underlying variable expressivity and partial penetrance may include unrecognized modifier genes, epigenetic influences, or environmental factors. Functional studies are needed to identify such modifiers and clarify their role.

Accurate molecular diagnosis could improve the outcome of patients and assist in prediction of disease prognosis, promote optional personalized treatment and avoid unnecessary treatments. In the context of *MAFB*-related disease Kaimori et al. (2021) described a positive effect of cyclosporine treatment in a patient with FSGS due to a *MAFB* variant [34]. Cyclosporine has a non-immunological effect on the stabilization of the actin cytoskeleton in kidney podocytes [35]. This could explain the direct effect of cyclosporine on reduction in protein excretion and its influence on synaptopodin, an actin binding protein that is an important regulator of podocyte function.

Given the frequent extra-renal manifestations associated with *MAFB* variants, multidisciplinary evaluation is essential, for example for early identification of hearing loss or ophthalmologic abnormalities. The molecular diagnosis also has implications for kidney transplantation. Whereas idiopathic FSGS is associated with a high risk of recurrence post-transplant, hereditary forms recur infrequently, and preemptive immunosuppression aimed at recurrence prevention is not required.

Improving diagnostic yield facilitates family-based testing, which is invaluable for identifying affected individuals and at-risk relatives. Furthermore, molecular diagnosis enables informed family planning, including evaluation of potential kidney donors and provision of reproductive counseling with options such as prenatal and preimplantation genetic testing.

## 5. Conclusions

In summary, we report a heterozygous *MAFB* variant, c.797T>C; p.(Leu266Pro), segregating in multiple related individuals from a single family, presenting with proteinuria, CKD, and variable extra-renal manifestations including DRS, auricular abnormalities, and neurodevelopmental involvement. We provide a comprehensive clinical description of a familial non-MCTO *MAFB*-associated phenotype and further expand the recognized clinical spectrum of *MAFB*-related disease. Structural modeling supports a domain-dependent pathogenic mechanism involving disruption of the C-terminal bZIP region. Further functional studies are required to elucidate the molecular mechanisms underlying this condition and refine genotype–phenotype correlations.

## Figures and Tables

**Table 1:** Clinical features of family members carrying the *MAFB* variant, demonstrating variable phenotypic expression. Abbreviations: CKD, chronic kidney disease; FSGS, focal segmental glomerulosclerosis; ASD, autism spectrum disorder; ADHD, attention-deficit/hyperactivity disorder; (-) not available.

Patient	Sex	Renal Manifestations		Extra Renal Manifestations			MAFB c.797T>C p.(Leu266Pro)
		Urinalysis	Renal Biopsy Findings	Ocular	Auricular	Developmental	
III1	F	Nephrotic range proteinuria; CKD	-	-	Bilateral hearing loss; common cavity malformation	Global developmental delay; ASD	Yes
III2	M	Nephrotic range proteinuria; CKD	-	Duane retraction syndrome	-	Developmental delay; ADHD	Yes
II3	F	Proteinuria	FSGS	-	-	normal	Yes
II4	F	-	FSGS	-		normal	-
II5	M	-	FSGS	-	-	normal	-
II6	F	Low grade proteinuria	-	-	-	normal	Yes

## Data Availability

The raw data supporting the conclusions of this article will be made available by the authors on request.

## Abbreviations

The following abbreviations are used in this manuscript:

CKD	chronic kidney disease
MAFB	MAF BZI Transcription Factor B
SRNS	steroid resistant nephrotic syndrome
MCTO	multicentric carpotarsal osteolysis syndrome
MRI	magnetic resonance imaging
ESKD	end-stage kidney disease
ES	Exome Sequencing
CNV	copy number variation
ACE	angiotensin-converting enzyme
eGFR	estimated glomerular filtration rate
ADHD	attention-deficit/hyperactivity disorder
ACMG	American College of Medical Genetics and Genomics
CMA	chromosomal microarray analysis
DRS	Duane retraction syndrome
CN	cranial nerve
